# Periductal Mastitis Occurring Among Hubble-Bubble Smokers: A Report of Two Cases and Literature Review

**DOI:** 10.7759/cureus.75686

**Published:** 2024-12-14

**Authors:** Rami Yaghan, Nehad M Ayoub, Lamees R Yaghan, Abdulla I Mohamed

**Affiliations:** 1 Department of Surgery, Arabian Gulf University, Manama, BHR; 2 Department of Surgery, Jordan University of Science and Technology, Irbid, JOR; 3 Department of Clinical Pharmacy, Jordan University of Science and Technology, Irbid, JOR; 4 Medical Skills and Simulation Center, Arabian Gulf University, Manama, BHR

**Keywords:** hubble-bubble, mammary fistula, periductal mastitis, smoking, waterpipe

## Abstract

Periductal mastitis (PM) is a form of nonlactational mastitis. The clinical picture varies from mild periareolar inflammation to frank retroareolar abscess formation. A huge amount of literature is incriminating cigarette smoking as a major contributing factor to the etiology of PM, and cessation of smoking is essential for a successful treatment. However, a search in PubMed and Scopus did not reveal any reports regarding the occurrence of PM among hubble-bubble smokers. Herein, we report two cases of PM in relation to hubble-bubble smoking (HBS) and review the related literature. In addition to antibiotic and surgical treatment, cessation of HBS was essential for sustained remission of PM.

## Introduction

Periductal mastitis (PM) is a form of nonlactational mastitis [1,2]. Subareolar duct dilatation and periductal inflammation with a predominant plasma cell infiltrate are the characteristic pathological findings [1,2]. The exact etiology of PM is still undetermined. Duct dilation is mostly a result of obstruction of a lactiferous duct by keratin plugs resulting from squamous ductal metaplasia [3]. Congenital nipple retractions are other possible contributing factors to duct blockage [1,3]. A damaged dilated duct is thought to be more prone to secondary infection, particularly anaerobic bacteria [1]. Cigarette smoking is a major contributing factor to the pathophysiology of PM although the exact mechanism is still under investigation [1,4,5]. Toxic tobacco smoking products are postulated to induce damage to the subareolar ducts, with tissue necrosis and subsequent infection [2]. The concentration of cotinine, a nicotine derivative, inside the ductal breast cells was found to be higher than its concentration in the plasma [2].

The clinical picture of PM ranges from superficial periareolar mastitis to frank retroareolar abscess formation that is occasionally complicated by a mammary fistula (Zuska’s disease) [1]. Treatment of PM consists of a combination of antibiotics and surgical intervention [1,4]. The cessation of smoking is an essential part of treatment [4,5].

Despite the huge amount of literature relating PM to cigarette smoking [1,3,4], there are no reports about the occurrence of PM in relation to hubble-bubble smoking (HBS). Worldwide, the popularity of HBS is increasing, particularly among the young age group and the female population, in contrast to the falling popularity of cigarette smoking [6,7]. Herein, we describe the clinical management of two cases of PM occurring in HBS. This will help in raising awareness among the medical community and the general population about the potential adverse effects of HBS on breast health in addition to its well-documented adverse effects on other body systems.

## Case presentation

Case 1

A single 19-year-old female patient presented with a painful right-sided retroareolar breast mass of two-week duration. The patient gave no history of pus or blood discharge from her nipple. The patient gave a history of daily HBS for the last two years. The average daily duration of HBS was two hours. She did not smoke cigarettes.

Examination of the right breast revealed a tender 3 × 3 cm retroareolar mass. The mass was tense and fixed to the base of the areola. The nipple was not inverted. There was mild redness at the lower part of the areola. Examination of the left breast and both axillae was normal. Breast ultrasonography revealed an irregular hypoechoic mass in the subareolar region with no circumscribed edges. The clinical diagnosis of PM with subareolar abscess formation was made.

**Figure 1:**
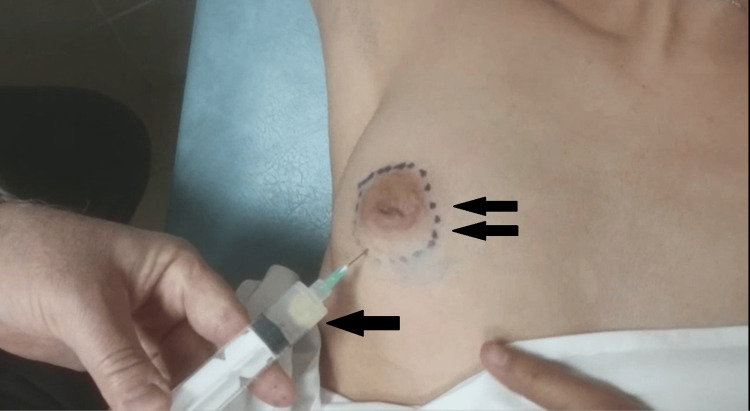
Periductal mastitis in a patient with history of hubble-bubble smoking This 19-year-old female patient, with a history of daily hubble-bubble smoking presented with a right-sided subareolar mass (double arrows). The diagnosis of periductal mastitis with breast abscess was made. Aspiration revealed thick pus that was evacuated by multiple aspirations (single arrow). The dotted markings were used for the clinical follow up of the response to treatment

Aspiration under local anesthesia revealed thick pus (Figure 1). The contents of the abscess were evacuated by multiple aspirations. Gram stain and culture of the aspirate were negative. She received clindamycin 300 mg twice daily for one week. A follow-up in the clinic showed the resolution of her disease. The patient was advised to quit HBS, but she was not compliant with the advice.

Two months later, she came again with a smaller abscess at the same site. She was treated by repeated aspirations followed by daily ductal irrigation with normal saline for one week till full clinical recovery. At this point, the patient stopped HBS. There were no further complaints over the next two months, and the patient was discharged from the clinic.

Case 2

A single 26-year-old female patient presented with severe pain in the right nipple of five-day duration. The patient gave no history of pus or blood discharge from her nipple.

The patient gave a history of HBS for the last five years. The frequency of HBS was three to four times per week with an average duration of two hours for each smoking session. She did not smoke cigarettes.

Examination of the right breast revealed redness and tenderness of the areolar and periareolar skin. No nipple inversion was noted. No masses were felt. Examination of the left breast and both axillae was normal. Breast ultrasonography was normal. The clinical diagnosis of PM was made. Treatment with clindamycin 300 mg twice daily for one week with cessation of HBS resulted in complete remission. Table 1 summarizes the clinical picture of the two patients.

**Table 1:** Clinical characteristics of two patients with periductal mastitis and hubble-bubble smoking

Characteristic	Case 1	Case 2
Age, years	19	26
Sex	Female	Female
Side	Right	Right
Duration of hubble-bubble smoking, years	2	5
Clinical presentation	Tender retroareolar mass	Periareolar redness and tenderness
Duration of symptoms, days	14	5
Breast ultrasound	Irregular hypoechoic mass	Normal
Treatment	Multiple aspirations and clindamycin	Clindamycin
Recurrence	Yes	No
Compliance with cessation of hubble-bubble smoking	Yes	No

## Discussion

This is the first report to suggest an association between PM and HBS. Patient 1 suffered from an indolent PM and experienced recurrent retroareolar abscess formation. Sustained remission was achieved after the patient stopped HBS. In patient 2, the clinical diagnosis of PM was made at an early presuppurative stage. Quick remission was achieved by treatment with clindamycin and cessation of HBS.

**Figure 2:**
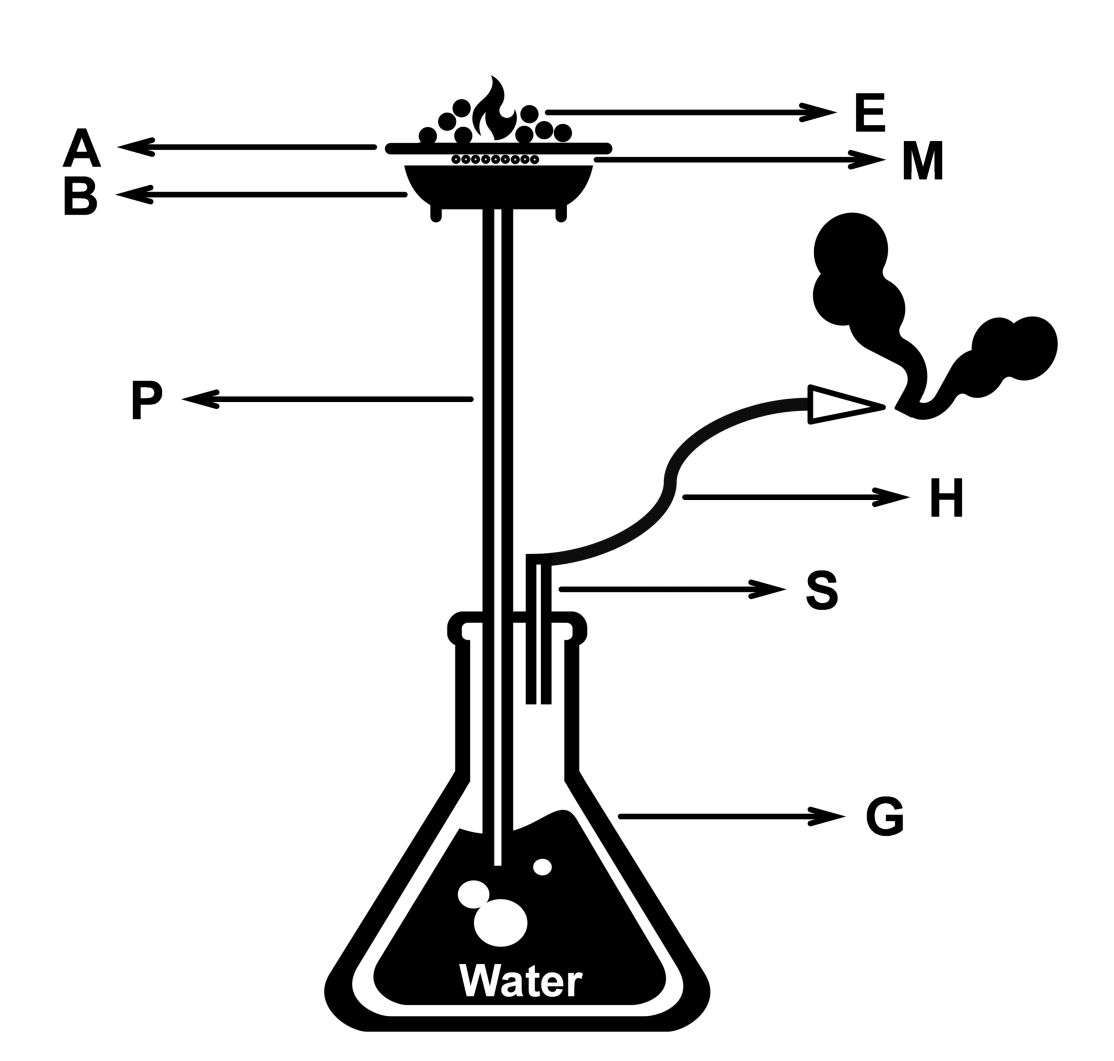
The basic structure of the hubble-bubble smoking device Moassal (M) which is a mixture of tobacco, fruit flavors, molasses, and glycerin is placed in a clay bowl (B) fixed to the upper end of a long pipe (P) and covered by a cribriform aluminum foil (A). The lower end of P is immersed in a water-filled glass container (G). A hose (H) is cabled to another small pipe (S) that is communicating to the empty part of G. Tobacco is burned by ember (E) placed on the top of A. The resultant smoke is sucked through P and filtered in the water by the negative pressure created at the free end of H by the inhaling smoker This figure is the original work of authors

Figure 2 illustrates the basic structure and function of the HBS device (also referred to in medical literature as waterpipe, narghile, argileh, sheesha, and hookah). Decorative styles and minor modifications in the construction parts vary according to the cultural heritage of each country. Different additives (among which are fruit flavors, molasses, glycerin, and honey) might be mixed with the tobacco leading to the different available brands of HBS preparations or the so-called moassal. 

The mechanism by which HBS might contribute to the pathophysiology of PM is most likely the same as in cigarette smoking and is related to the absorption of a toxic product resulting from tobacco burning [1,2,4,5]. However, the fact that various combinations of additives (fruit flavors, molasses, glycerin, and honey) are added to the HBS preparations might widen the spectrum of the absorbed toxins that might contribute to tissue damage. 

There is a misconception by the general population that the passage of smoke through water before inhalation extracts most of the resultant toxic products, making HBS a safer option than cigarette smoking [6]. This, in addition to the social acceptability of HBS and the enjoyment of the extra flavors, adds to the increasing popularity of HBS among women [6,7]. 

HBS induces acute cardiorespiratory adverse effects including increased heart rate, blood pressure, respiratory rate, and carbon monoxide (CO) level [8]. Chronic HBS is associated with coronary artery disease, chronic increase in CO level, altered pulmonary function tests, and chronic obstructive pulmonary disease. Associations with lung cancer, upper gastrointestinal cancers, bladder cancer, and potentially oral cancers are widely addressed in the literature [8]. Further reported adverse effects of HBS on women’s reproductive health include early weaning of their children, a reduced birth weight, and increased mortality of their infants [8,9].

There is evidence for a moderate increased risk of breast cancer in women who smoke tobacco or are exposed to passive smoking [10]. In relation to this, HBS was found to induce epithelial to mesenchymal transition of MCF7 and BT20 breast cancer cell lines, enhancing the invasion ability of these cells [11]. Furthermore, HBS was reported to provoke a downregulation of E-cadherin and an upregulation of focal adhesion kinase, which are key regulators of cancer progression genes [11].

The treatment of PM is sometimes difficult with a relatively high rate of recurrence [4]. The treatment modality depends on the extent of the disease and the chronicity of symptoms. In the early pre-purulent stages of PM (case 2), antibiotic treatment in addition to quitting smoking is usually enough [1-3]. A combination of antibiotic and surgical treatment is the most widely adopted treatment for established purulent stages [2].

Empirical antibiotic treatment is usually directed against *Streptococci*, *Staphylococci*, and anaerobes [12]. Antibiotic modification depends on the clinical response and the culture and sensitivity results. The involved anaerobic bacteria are difficult to culture and require special sampling and culture techniques. However, the treating surgeon should consider the presence of anaerobic infection even if the cultures are negative as was the case in this patient 1 [4-8]. The type of surgical intervention depends on the PM stage. It ranges from simple aspirations, duct irrigation, incision and drainage, and minor lesion excision to total ductal excision with plastic corrective techniques to minimize the resultant areolar depression [1,4,5,12].

Cessation of smoking is essential for successful treatment, particularly at the stage of chronic abscess and fistula formation [4,5]. PM recurrence in patient 1 was mostly predisposed to by the continuation of HBS and not an inadequacy of the opted surgical treatment.

As demonstrated by our two cases, the clinical features and the treatment principles of PM in HBS do not seem to depart from those of PM occurring in cigarette smoking. However, a lack of awareness about the potential adverse effects of HBS on breast health issues might explain the paucity of reports in this regard. 

## Conclusions

We described two cases of PM occurring in HBS. Most likely, the underlying pathophysiology mimics the pathophysiology of PM occurring in cigarette smoking. Raising awareness about the potential adverse effects of HBS on women’s breast health issues, in addition to its well-documented adverse effects on other body systems, might aid the intervention protocols against HBS.
